# Calcineurin

**DOI:** 10.1186/s12964-020-00636-4

**Published:** 2020-08-28

**Authors:** Trevor P. Creamer

**Affiliations:** Center for Structural Biology, Department of Molecular & Cellular Biochemistry, 741 S. Limestone Street, Lexington, KY 40536-0509 USA

**Keywords:** Calcineurin, Calmodulin, Calcium, Intrinsically-disordered region, Transcription, Receptors, Channels, Mitochondria, Microtubules

## Abstract

The serine/threonine phosphatase calcineurin acts as a crucial connection between calcium signaling the phosphorylation states of numerous important substrates. These substrates include, but are not limited to, transcription factors, receptors and channels, proteins associated with mitochondria, and proteins associated with microtubules. Calcineurin is activated by increases in intracellular calcium concentrations, a process that requires the calcium sensing protein calmodulin binding to an intrinsically disordered regulatory domain in the phosphatase. Despite having been studied for around four decades, the activation of calcineurin is not fully understood. This review largely focuses on what is known about the activation process and highlights aspects that are currently not understood.

Video abstract

Video abstract

## Background

The serine/threonine phosphatase calcineurin (CaN) is activated by increased intracellular calcium concentrations [1–4]. Along with the calmodulin-activated kinases [5], CaN directly links calcium signaling to protein phosphorylation states and plays an essential role in numerous signaling processes [6]. CaN is found in eukaryotes and is conserved from single cell organisms through to *Homo sapiens* [2, 7]. Although this review deals primarily with the human isoforms, much of what is covered should be applicable to other organisms.

This review will largely focus on how calcium acts to activate CaN but will start with a brief introduction to how CaN is coupled to calcium signaling, and to some of the phosphatase’s substrates and the signaling processes of which they are a part (summarized in Table 1). This should not be taken as an exhaustive list of substrates nor a definitive description of how CaN modulates their activities. Indeed, how CaN recognizes its substrates is an active area of study, and there are ongoing efforts to identify and characterize more CaN substrates [32, 33].

**Table 1:** Signaling processes that calcineurin plays roles in and example substrates

Signaling processes/molecules	Example calcineurin substrates
Transcription	NFATs (nuclear factors of activated T-cells) [3, 4, 8–16] FOXO (forkhead transcription factors) [17, 18] MEF2 (myocyte-specific enhancer factor 2) [13, 15, 16] TFEB (transcription factor EB) [12, 16, 18, 19]
Receptors and channels	AMPARs (α-amino-3-hydroxy-5-methyl-4-isoxazole-propionic acid receptors) [20, 21] NMDARs (NMDA-type ionotropic glutamate receptors) [20, 21] TRESK (tandem-pore-domain weakly inward rectifying potassium channels (TWIK)-related spinal cord potassium channels) [22–24] NHE1 (Na+/H+ exchanger 1) [16, 25, 26]
Mitochondria	DRP1 (dynamin-related protein 1) [27] BAD (Bcl-2/Bcl-XL-antagonist, causing cell death) [28–30]
Microtubules	tau [31] MAP2 [31]

### Signaling

#### Calcium signaling, calcineurin, and calmodulin

CaN is activated in response to increases in calcium concentrations in the cell [1, 2, 34]. The molecular details of the activation process will be discussed in detail below, but in short both CaN and the calcium-sensing protein calmodulin (CaM) bind calcium, with CaM then binding to CaN. The CaM:CaN complex forms the active phosphatase. This process directly couples calcium signaling to dephosphorylation in much the same way that calcium signaling is coupled to phosphorylation through the CaM-modulated kinases [5]. One notable difference however is that there are multiple CaM-modulated kinases but CaN is the only phosphatase known to be directly activated by calcium.

A brief discussion of CaM is warranted. This calcium-sensing protein plays critical roles in numerous processes and is known to have in excess of 200 different substrates [35, 36]. These encompass a broad range of proteins and enzymes. These include, but are not limited to, the previously-mentioned the CaM-modulated kinases [5], nitric oxide synthases [37], the calcium-release channel ryanodine receptors [38], proteins such as neurogranin and growth-associated protein-43 [39], and even the microtubule-associated protein tau [40, 41]. Notably, CaM modulates the activities of enzymes such as CaN and the CaM-modulated kinases which are involved in numerous signaling processes.

Interestingly it has been suggested by Persechini and Stemmer that CaM concentrations in cells are limiting [42]. In other words, there are more potential substrate molecules than CaM molecules available to bind to them. The concentration of CaM in mammalian cells has been estimated to be around 5 μM [43]. As noted by Persechini and Stemmer, this concentration is comparable to both the high end of calcium concentrations and the concentrations of some substrates [42]. As noted by these authors, CaM activity will thus be regulated by its proximity to substrate and released calcium, plus substrate affinity.

CaN is primarily located in the cytosol [2]. A-kinase anchoring protein-79 is known to bind inactive CaN and localize it to the plasma membrane in the vicinity of calcium channels [44–46]. This would put CaN in a favorable location for binding CaM upon increases in intracellular calcium concentration. Furthermore, CaM’s affinity for CaN is in the low picomolar range, making this the tightest known CaM:substrate binding [47–49]. Taken together, these observations suggest that CaN is a particularly important CaM substrate and that downstream targets of CaN are also of some importance. Some of these downstream targets will now be discussed.

#### Transcription factors

The most studied substrates of CaN are the family of nuclear factors of activated T-cells (NFATs) [3, 4, 8]. The desphosphorylation of an NFAT in the cytosol results in exposure of a cryptic nuclear localization signal, resulting in the NFAT moving into the nucleus and activating a variety of transcription programs. Perhaps the most well-known of these are processes that result in activation of T cells within the immune system [9, 10]. For this reason, the immunosuppressant drugs tacrolimus and cyclosporin are used to bind to and inhibit CaN [33]. The NFATs dephosphorylated by CaN activate transcription programs in other systems such as neurons and astrocytes [11, 12], as well as the heart and skeletal muscle [13–16].

Other transcription factors are regulated by dephosphorylation by CaN. CaN dephosphorylates the forkhead transcription factors (FOXO) involved in metabolism and autophagy [17, 18]. Other transcription factor substrates include the myocyte-specific enhancer factor 2 (MEF2) [13, 15, 16] and transcription factor EB (TFEB) [12, 16, 18, 19]. The latter two transcription factors regulate a wide variety of processes including cardiac function, neuronal function, and autophagy.

#### Receptors and channels

CaN is also known to interact with and help regulate the activity of several receptors and ion channels. These in turn control a variety of signaling pathways. CaN dephosphorylates the α-amino-3-hydroxy-5-methyl-4-isoxazole-propionic acid receptors (AMPARs) and the NMDA-type ionotropic glutamate receptors (NMDARs), playing crucial roles in synaptic long term potentiation and long term depression [20, 21]. It is also involved in modulating the activity of tandem-pore-domain weakly inward rectifying potassium channels (TWIK)-related spinal cord potassium (TRESK) channels in the central nervous system as well as in immunological response [22–24]. CaN has also been identified as an important regulator of the Na+/H+ exchanger 1 (NHE1) [16, 25, 26].

#### Mitochondria associated proteins

CaN has been linked with a number of proteins associated with mitochondria for quite some time [27, 28, 50]. For example, CaN dephosphorylates dynamin-related protein 1 (DRP1), linking catecholamines, via calcium signaling, to mitochondrial fission [27]. In addition, apoptosis initiated by sustained increases in intracellular calcium concentrations occurs via CaN dephosphorylation of the protein BAD (Bcl-2/Bcl-XL-antagonist, causing cell death) [28–30].

#### Microtubule-associated proteins

Calcium signaling is linked to neuronal cytoskeletal dynamics, and neurodegeneration, in part through CaN [31]. CaN is one of the phosphatases known to dephosphorylate the microtubule-associated proteins tau and MAP2. The phosphorylation state of these proteins controls their binding to and stabilization of microtubules. The phosphorylation state of tau also controls its association with stress granules within neurons [51].

### Structure and function

#### CaN structure

**Fig. 1:**
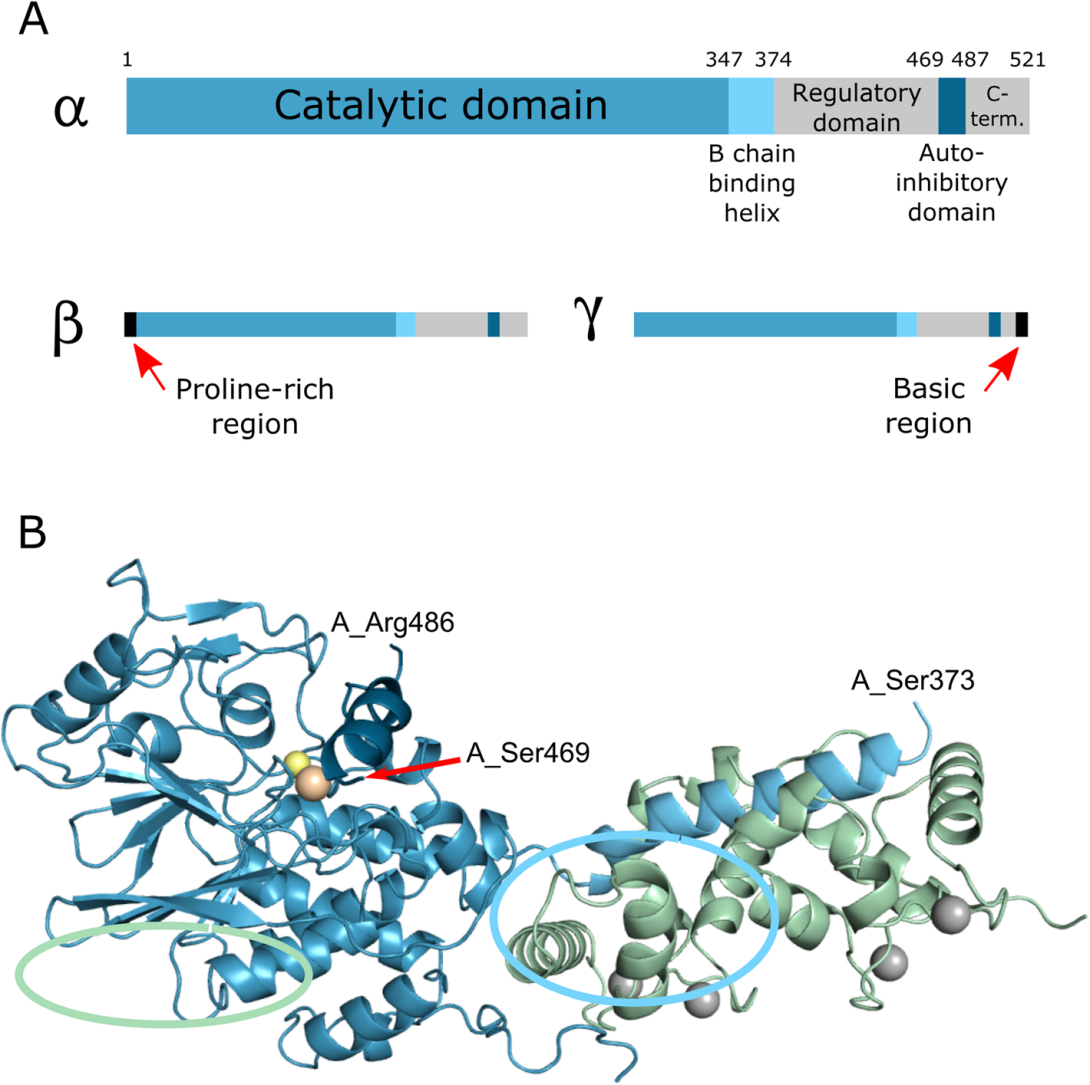
**a**. Domain structures of the CaN A chains from the three human isoforms. The residue numbering for αCaN was taken from the crystal structure (PDBID 1AUI [55];). For the β and γ isoforms, the regions of greatest difference with the α isoform are highlighted. **b**. Crystal structure of human αCaN (PDBID 1AUI [55];). The A chain is colored blue and the B chain green. The orange and yellow spheres denote a Zn-Fe center in the active site in the catalytic domain and the grey spheres denote Ca^2+^ bound to the B chain. Residues within the A chain between and beyond which electron density is not detected are indicated. The green oval denotes the location of the PxIxIT substrate binding site and the blue oval the LxVP substrate binding site

Calcineurin (CaN) is a heterodimer expressed as three different isoforms; αCaN, βCaN and γCaN [1–3]. The overall domain and three-dimensional structure of each isoform is much the same with a catalytic A chain (CnA) and calcium-binding regulatory B chain. The ~ 58–64 kDa A consists of the catalytic domain, a B chain-binding helix, the regulatory domain, an autoinhibitory domain, and an unstructured C-terminal domain of unknown function (Fig. 1a). The ~ 19 kDa B chain (CnB) is structurally homologous to calmodulin (CaM) and consists of two calcium-binding lobes connected by a short linker. Each lobe of CnB consists of two calcium-binding EF hands [1, 52–54].

The crystal structure of the calcium-loaded human αCaN (PDB ID 1AUI [55];) is shown in Fig. 1b. Notably, the 96-residue regulatory domain and 35-residue C-terminal domain are not seen in the structure due to a lack of electron density. This suggests that these domains occupy multiple conformations in the crystal. Multiple structures of intact CaN, all in the calcium-bound state, have been deposited in the PDB with the regulatory and C-terminal domains remaining unseen in all [26, 56–60].

Depictions of the domain composition of each of the three isoforms of CaN are given in Fig. 1a. The α isoform of CaN was the first identified, is the simplest, and is the dominant form in neurons [1–3]. It consists of the αCnA and CnB1 chains. The β isoform is the dominant form in most non-neuronal cell types and consists of βCnA and CnB1 chains. The major difference between βCnA and αCnA is an N-terminal proline-rich extension, including a run of ten consecutive prolines, on the former isoform. Little is known about this extension although there is some evidence that it is involved in modulating substrate specificity [61]. The γ isoform consists of γCnA and CnB2 chains. This isoform was initially thought to be testes specific but has more recently been found to be more broadly distributed, albeit at low levels, and has been implicated as playing a role in schizophrenia [62–64]. γCnA differs from αCnA largely by a basic residue-rich region at the end of the C-terminal domain [1–3]. The function of this region is currently unknown. The human CnB1 and CnB2 isoforms differ by just three residues in length, have 83% sequence identity, and appear to function identically.

#### Disorder within CaN

**Fig. 2:**
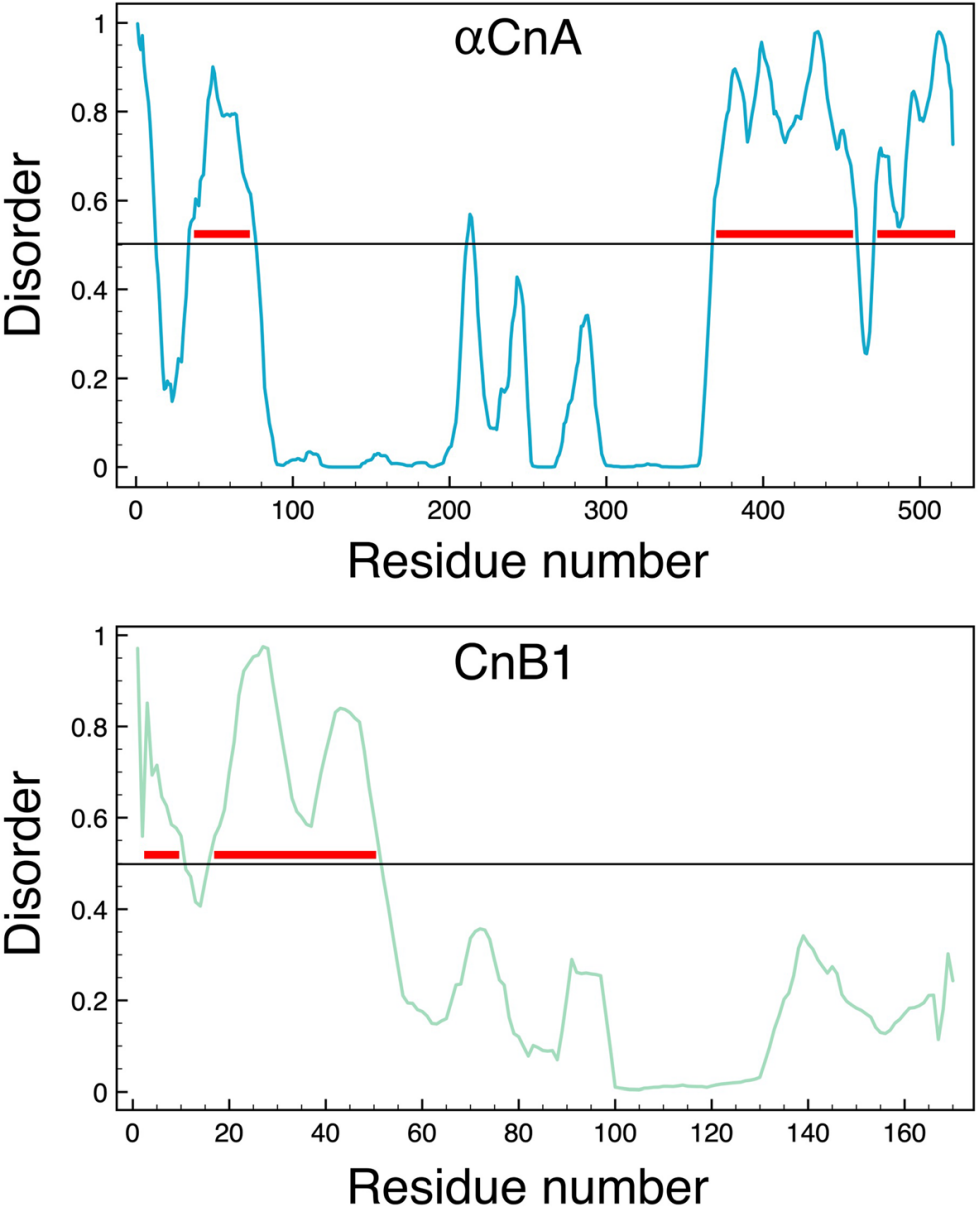
Disorder predictions for the human αCnA (top panel) and CnB1 (bottom panel) chains. Values above 0.5 denote predicted disorder. Major regions of predicted disordered are marked with red bars

As noted above, the 96-residue regulatory and 35-residue C-terminal domains of the αCnA chain are not observed in crystal structures of CaN (see Fig. 1b). There are an additional thirteen residues at the N-terminus of this chain that are also not observed, along with four residues of the N-terminus of the CnB1 chain. Predictions from the PONDR VLXT disorder predictor [65, 66] for the human αCnA and CnB1 chains are shown in Fig. 2. Regions with scores above 0.5 are predicted to be disordered. Major regions of predicted disorder are denoted with red bars in Fig. 2. Interestingly there is a region between residues 34 and 76 in αCnA that is predicted to be disordered but is clearly a well-ordered segment of the catalytic domain in known CaN crystal structures. Aside from that region, two others in αCnA are strongly predicted to be disordered. These are the regulatory and C-terminal domains. The short region of predicted order between these two disordered regions corresponds to the start of the autoinhibitory domain.

A significant portion of the N-terminal lobe of CnB1 is predicted to be disordered (Fig. 2), and yet is clearly well-folded in the crystal structure (Fig. 1b). As is discussed below this lobe of CnB1 is thought to be dynamic in the activation process.

It is not clear what role the 35-residue C-terminal tail of the αCnA chain might play. As will be discussed below, the regulatory domain is an important component in the activation of CaN, with the disorder within that domain playing an essential role.

#### Substrate binding

Substrates do not appear to bind directly in the CaN active site cleft. Rather, there are two substrate binding sites on the surface of the phosphatase. These are named the PxIxIT and LxVP sites based on the consensus binding motifs [4, 32, 33, 58, 67–70]. Many known CaN substrates possess both motifs with the sites to be dephosphorylated generally located between the two, although there are some substrates with just one binding motif [45]. The PxIxIT site is located on the side of the catalytic domain and is always accessible (green oval in Fig. 1b) [58, 59, 71, 72]. Sequences that bind to this site bind directly to an edge strand of a β-sheet, acting as an additional strand.

The second binding site, LxVP, is located at the interface of the B chain binding helix in the A chain and the C-terminal lobe of the B chain (blue oval in Fig. 1b) [4, 58, 69, 71, 73]. The well-known immunosuppressant drugs cyclosporin and tacrolimus (FK506) bind in this region and inhibit substrate binding [55, 74, 75]. LxVP is actually something of a misnomer for the binding motif. It has been shown that a polar/aromatic residue pair are favored immediately before the leucine, leading to the suggestion that the motif should more accurately be called πϕLxVP [60]. Notably, the LxVP binding site is only accessible when CaN is fully active.

### Activation

**Fig. 3:**
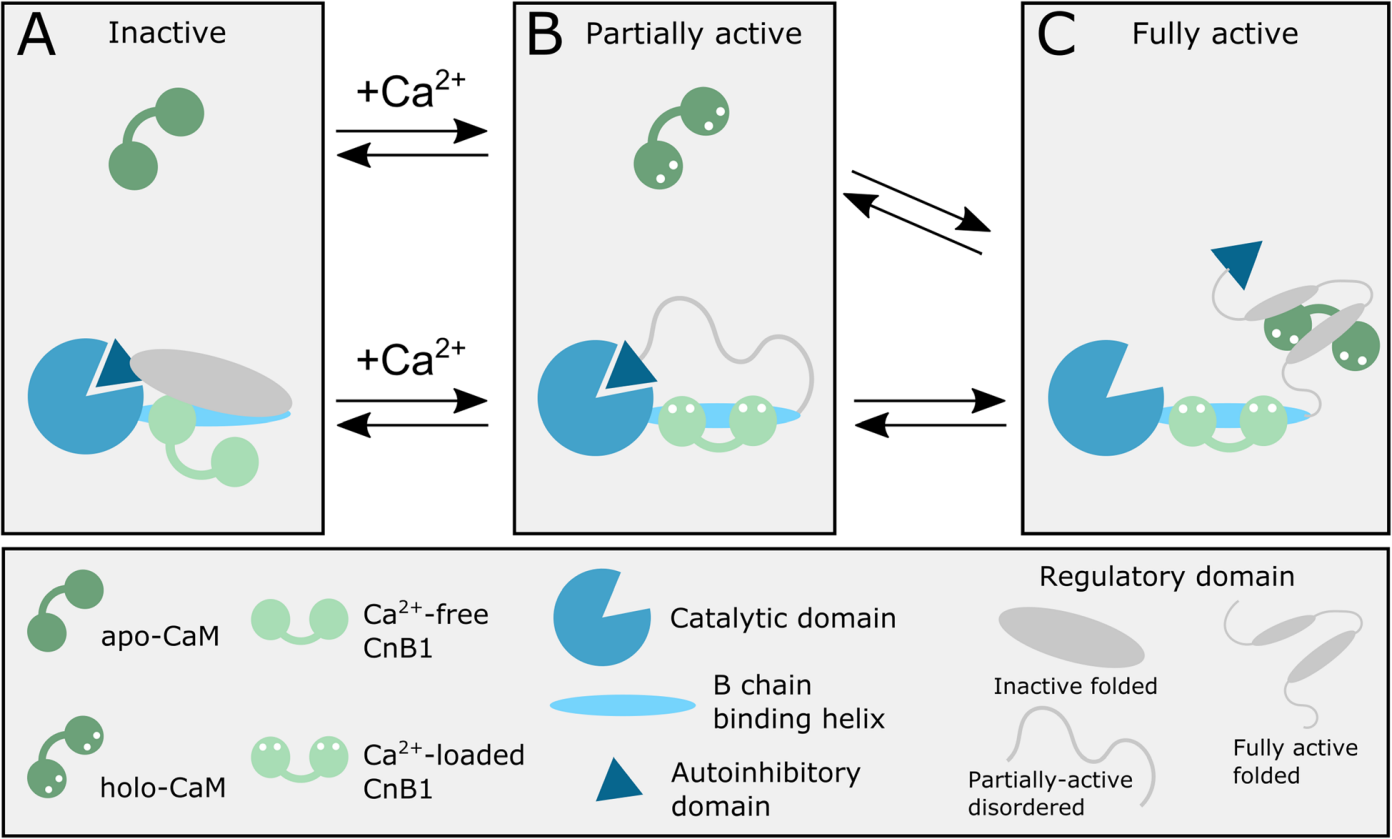
Proposed models for the (**a)** inactive, (**b)** partially active, and **c**. fully active states of CaN. The inactive state (**a**) occurs at basal calcium concentrations and is thought to have the regulatory domain folded onto the B chain binding helix and the N-terminal lobe of CnB1 unbound. The partially active state (**b**) occurs when calcium is bound to CnB1 but prior to CaM binding. In this state the N-terminal lobe of CnB1 binds to the B chain binding helix, releasing the regulatory domain into a disordered state. The fully active state (**c**) occurs when holo-CaM has bound to CaN causing the regulatory domain to fold and removing the autoinhbitory domain from the active site

It has been known for some time that CaN is activated in response to increased intracellular calcium concentrations and that full activation requires the calcium-sensing protein calmodulin (CaM) [1–3, 34]. A detailed description of the full activation process is not available, but it does appear to involve three distinct states (Fig. 3). What is known has been determined largely through study of the αCaN isoform, but there are no compelling reasons to believe the activation processes of the βCaN and γCaN isoforms differ significantly.

#### Inactive CaN

The inactive state of CaN (Fig. 3a) exists at basal intracellular calcium levels which have been estimated to be around 100 nM [76]. Much of what we know about the inactive state was determined by the groups of Klee [52, 53, 77–79] and Perrino [80, 81]. Klee and co-workers demonstrated that both the regulatory domain of the αCnA chain and the linker between the two lobes of the CnB1 chain were susceptible to cleavage in limited proteolysis experiments in calcium-dependent manners [52, 77, 78]. In the absence of calcium, or at low concentrations, the regulatory domain is protected from proteolysis, whereas at high calcium concentrations, but in the absence of CaM, it is readily cleaved into multiple small fragments. This observation suggests that the regulatory domain is folded at basal calcium levels and becomes unfolded, or disordered, when calcium concentrations increase. In contrast, at low calcium concentrations the linker connecting the two calcium-binding lobes of CnB1 is cleaved in limited proteolysis but becomes protected at higher concentrations. Furthermore, when CnB1 is cleaved at low calcium concentrations the N-terminal lobe of the chain is released while the C-terminal lobe remains tightly bound to the αCnA chain.

Little is known about the calcium-binding properties of CnB1. Gallagher et al. determined the calcium affinities for the isolated CnB1 chain and found the two sites in the N-terminal lobe had affinities in the 11–81 μM range and the two in the C-terminal lobe in the 30–150 nM range [82]. These affinities would support the idea that the N-terminal lobe does not have calcium bound at basal intracellular concentrations but that the C-terminal lobe might have one or even two calcium ions bound. It’s important to note however that these affinities will be altered when CnB1 is bound to the αCnA chain.

Perrino et al. [81] generated αCnA chain constructs where the chain was truncated either just before the CaM binding site within the regulatory domain or just after the CaM binding site and combined these with CnB1. The former truncation resulted in an αCaN that was largely active against a substrate that bound to the LxVP binding site regardless of the calcium concentration. The latter truncated αCaN however required both elevated calcium and the presence of CaM to be fully active. This suggests that the CaM binding site within the regulatory domain contains elements that inhibit substrate binding to the LxVP site at the interface formed by the B chain binding helix in the A chain and the C-terminal lobe of CnB1. Others have since published evidence that the regulatory domain contains autoinhibitory elements that function in conjunction with the autoinhibitory domain to keep CaN inactive at low calcium concentrations [57, 80, 83, 84].

Taken together the above observations lead to a model for the inactive state of αCaN where the N-terminal lobe of CnB1 is not bound to the B chain binding helix of the αCnA chain, likely due to that lobe being devoid of calcium (Fig. 3a) [1, 34, 82]. This allows the regulatory domain to be folded onto the B chain binding helix and the interface between that helix and the C-terminal lobe of CnB1. Under these conditions the autoinhibitory domain would be bound to the active site cleft of the catalytic domain keeping the phosphatase inactive (Fig. 3a).

#### Partially active CaN

A partially active state of αCaN is obtained when calcium concentrations are increased but CaM is not present [52, 53, 79, 85]. All published crystal structures of full-length human CaN are of this calcium-loaded form (e.g. 1AUI [55] as shown in Fig. 1b). Each lobe of CnB1 has two calcium ions bound, and the N-terminal lobe is bound to the B chain binding helix in the A chain. The regulatory domain of the A chain is not seen in these structures and has been shown to be readily cleaved in limited proteolysis experiments [52, 77, 78]. Of note, in those proteolysis experiments CnB1 was not susceptible to cleavage when fully calcium loaded. Although the autoinhibitory domain is bound in the active site cleft in the crystal structures of the calcium-loaded form of CaN, in vitro experiments have demonstrated that this form is somewhat active [52, 53, 79, 85]. This suggests that the autoinhibitory domain is not tightly bound in this state.

The lack of electron density for the 96-residue regulatory domain in CaN crystal structures, and its vulnerability to proteolysis, spurred Dunker and co-workers to consider that this domain might be devoid of structure and to subsequently develop predictors of protein disorder [65, 86]. Creamer and co-workers have used a variety of techniques to probe the conformational properties of the regulatory domain in calcium-loaded human αCaN [34, 87–91]. A combination of circular dichroism (CD) spectrapolarimetry, fluorescence spectroscopy, hydrogen-deuterium exchange mass spectrometry (HXMS), and limited tryptic digestion was used to demonstrate that the regulatory domain was indeed intrinsically disordered in the partially active form of αCaN [87, 88]. Later, in collaboration with the Fitzkee laboratory, NMR was employed to confirm these findings [91].

In Fourier transform infrared spectroscopy (FTIR) experiments, Fu et al. [92] found the regulatory domain of CaN to exist in a very dynamic state supporting the idea that it is intrinsically disordered.

The above observations suggest the model for partially active CaN illustrated in Fig. 3b. The increased calcium concentration leads to both lobes of CnB1 becoming fully loaded with the metal ion. The N-terminal lobe of CnB1 binds to the B chain binding helix, causing the regulatory domain to unfold and become disordered. The disordered regulatory domain at least partially exposes the previously occluded LxVP site, allowing substrate to bind. The unfolding of the regulatory domain also presumably weakens the binding of the autoinhibitory domain in the active site cleft of the catalytic domain allowing for partial activity.

#### Fully active CaN

**Fig. 4:**
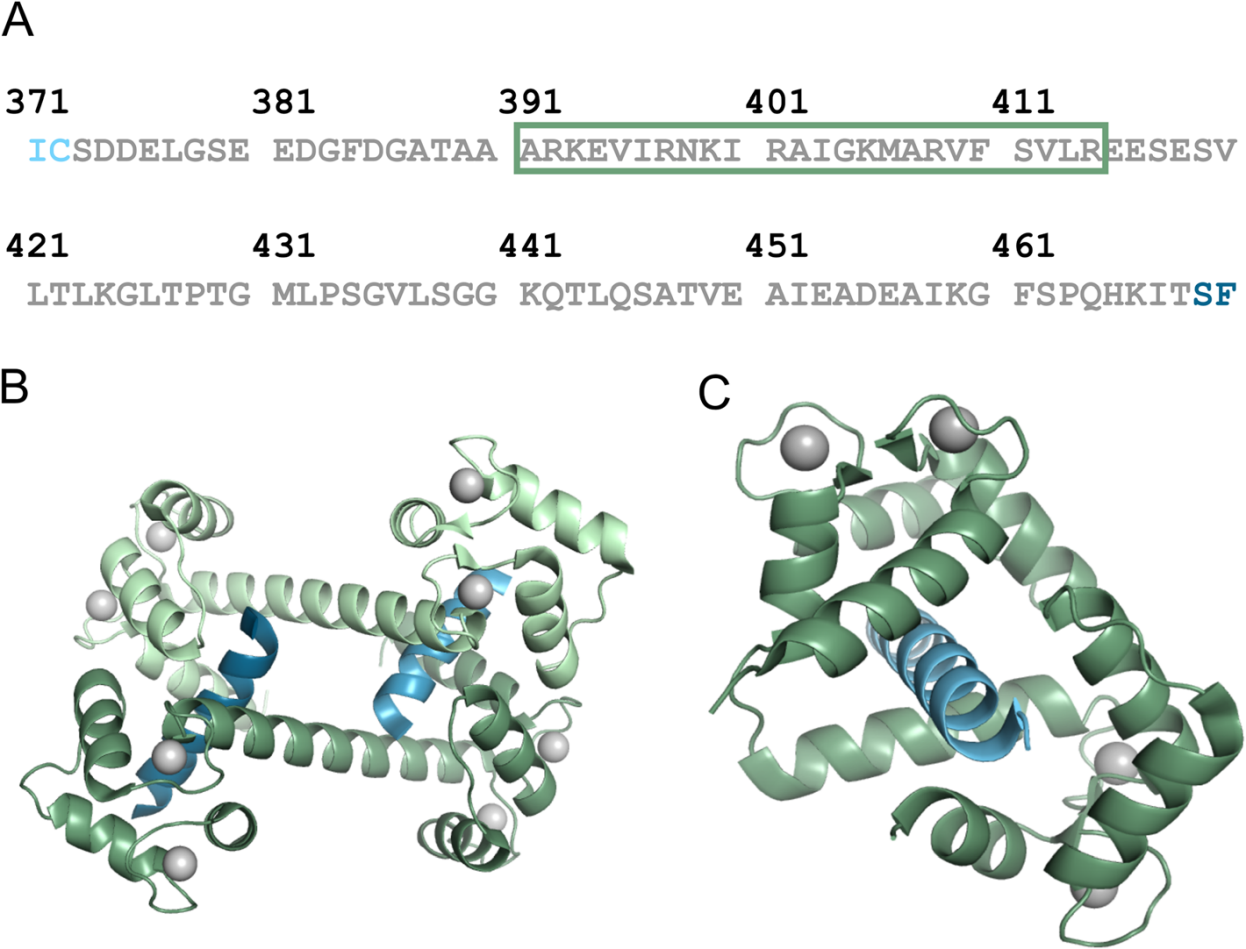
a. Sequence of the regulatory domain (regulatory domain) from the human αCaN A chain with the CaM binding region denoted by the green box. **b**. 2:2 stoichiometric structure of holo-CaM bound to the CaM binding region from αCaN (PDBID 2R28 [93, 94];). Holo-CaM is colored green, the CaM binding region blue, and the grey spheres are Ca^2+^. **C.** 1:1 stoichiometric structure of holo-CaM bound to the CaM binding region from αCaN (PDBID 4Q5U [89];)

Full activation of CaN requires both calcium and calcium-loaded CaM (holo-CaM) to bind to the phosphatase [1–3]. It has been known for some time that holo-CaM binds to a 24-residue region within the regulatory domain of the A chain (the CaM binding region; Fig. 4a) and that binding results in release of the autoinhibitory domain from the active site cleft. It has also been known for almost four decades that the regulatory domain in this fully active state is conformationally distinct from that in the partially active state. This was determined by Klee and co-workers in their limited proteolysis experiments [52, 77, 78]. These workers found that the regulatory domain, which is readily cleaved in partially active CaN, is protected from proteolysis when holo-CaM is bound in the fully active state.

There was some controversy over how CaM bound to its CaM binding region in CaN. Jia and co-workers published crystal structures of holo-CaM bound to the CaN CaM binding region that had two CaM molecules sharing binding to two CaN CaM binding region peptides (Fig. 4b) [93, 94]. Majava and Kursula solved a similar crystal structure, but went on the perform extensive hydrodynamic experiments including small-angle X-ray scattering and size-exclusion chromatography that indicated the stoichiometry of the CaM:CaM binding region peptide complex was 1:1 in solution [95]. In their first paper on the 2:2 structure, Ye et al. also presented gel filtration data suggesting the complex predominantly was 1:1 in solution [93]. O’Donnell et al. used multiple hydrodynamic measurements to determine that holo-CaM bound to the CaM binding region from βCaN with a 1:1 stoichiometry [48]. In later work, Jia and co-workers performed small-angle X-ray scattering on holo-CaM bound to the regulatory domain from αCaN and found they could only fit a 1:1 complex to the resulting envelop [56]. Dunlap et al. solved the crystal structure of holo-CaM bound to the CaM binding region from αCaN and obtained a 1:1 complex (Fig. 4c) [89]. These authors used size-exclusion chromatography and rotational correlation coefficients determined using time-resolved fluorescence spectroscopy to show the holo-CaM:CaM binding region complex had an almost entirely 1:1 stoichiometry in solution. The overwhelming majority of the evidence thus points to a single holo-CaM binding to a single CaN CaM binding region.

Creamer and co-workers have noted that holo-CaM binding to its known 24-residue binding region within the regulatory domain would not alone be sufficient to protect the 96-residue domain from proteolysis [34, 87–90]. There are, for example, multiple trypsin cleavage sites (basic residues) within the regulatory domain but outside the CaM binding site (Fig. 4a). The CaM binding region folding into an α-helix is also unlikely to be sufficient to remove the autoinhibitory domain from the active site cleft given there are around 55 residues between the end of the CaM binding region and the start of the autoinhibitory domain.

A HXMS experiment involving holo-CaM binding to an isolated regulatory domain construct indicated that a ~ 35-residue region of the regulatory domain C-terminal to the CaM binding region was somewhat protected from exchange suggesting that this region became structured upon binding [87]. Careful analysis of CD spectra led to the suggestion that a second, distal helical region formed in the regulatory domain upon holo-CaM binding and that this distal helix was folded onto the surface of CaM. In their work on holo-CaM bound to the regulatory domain Jia and co-workers found they could fit an additional α-helix in the envelop obtained from small-angle X-ray scattering measurements [56].

By plotting the sequence on a helical wheel Dunlap et al. were able to determine the most likely region for this distal helix was residues 441 to 458 [88]. This stretch of sequence could potentially form an amphipathic helix. In order to test this, each of the three alanines on the putative hydrophobic face were individually mutated to glutamate. Each of these mutations reduced the helical content of the holo-CaM-bound regulatory domain but did not abolish binding. Furthermore, these mutations greatly reduced the activity of holo-CaM-bound full-length αCaN indicating that formation of the distal helix is crucial for full activation of the phosphatase [88].

The holo-CaM:regulatory domain complex has so far eluded extensive crystallization efforts by the Creamer laboratory. Yadav et al. used a ^15^N,^13^C-labeled construct to obtain NMR resonance assignments for the intrinsically disordered regulatory domain [91]. However it was found that the holo-CaM:regulatory domain complex was in the slow to intermediate exchange regime, precluding solution of a high resolution structure via NMR. It is possible that the distal helix region is very dynamic, making short-lived contacts with the surface of holo-CaM but maintaining sufficient structure to exhibit some protection in HXMS and helical CD signal as observed by Creamer and co-workers [87, 88].

In more recent work, Sun et al. employed a variety of computational approaches in an attempt to identify where the distal helix forms on the surface of holo-CaM [96]. Four putative regions of interaction were identified. The prediction from these computations was that a helix encompassing residues 30 through 40 in the N-terminal lobe of holo-CaM is the most likely site of interaction with the distal helix. This prediction has yet to be tested experimentally.

Based on the existing data, the current model for the fully active state of CaN has holo-CaM bound to the CaM binding region in the regulatory domain (Fig. 3c). A region of the regulatory domain C-terminal to the CaM binding region folds into an α-helix somewhere on the surface of holo-CaM. The resulting compaction of the 96-residue regulatory domain is sufficient to dislodge the autoinhibitory domain from the active site cleft of CaN leading to its full activation.

#### Distal helix stability

In their work on the distal helix in the regulatory domain of αCaN, Creamer and co-workers made the somewhat puzzling observation that this helix had a melting temperature (T_m_) of just ~ 38 °C in dilute buffer [90]. The distal helix would be, on average, unfolded and holo-CaM-bound αCaN presumably not be fully active nearly half of the time under such circumstances. Of course, conditions inside a cell are very different to the dilute solutions employed in the laboratory. Cook and Creamer were able to show that the T_m_ of the distal helix increased to ~ 43 °C in solutions containing 200 g/L dextran 70 or ficoll 70, conditions that mimic the crowded interior of cells [90]. Furthermore, the activity of αCaN was increased in the presence of these crowding agents but not in the presence of an equivalent concentration of sucrose. These observations suggest that crowding agents increase the stability of distal helix and that holo-CaM-bound αCaN would be fully active in cells at normal physiological temperature.

The observed T_m_ of ~ 43 °C for the distal helix under cell-like conditions indicates it is just barely stable and that αCaN activity would be sensitive to relatively small increases in temperature [90]. This led to the speculation that perhaps this temperature sensitivity may have evolved as a mechanism for controlling fever response. CaN is known to be involved in the control of inflammation and perhaps fever [97–99]. It has been shown that expression of Rcan1 (regulator of calcineurin 1), an endogenous inhibitor of CaN, reduces expression of pro-inflammatory genes [100]. Temperatures associated with high fever could elicit a similar response by destabilizing the distal helix thereby allowing the autoinhibitory domain to occupy the active site cleft of CaN.

It is also possible the marginal stability of the distal helix is an indicator of a dynamic nature as suggested in the previous section. Beyond the speculated control of fever and inflammation, the evolutionary advantage of a dynamic distal helix region is not clear.

#### Holo-CaM:CaN binding affinity

Hubbard and Klee initially determined that holo-CaM bound to αCaN with a K_D_ of ~ 16 nM making this one of the tightest CaM:substrate interactions [101]. Quintana et al. later used stopped-flow fluorescence measurements to study the binding of holo-CaM to αCaN [47]. They measured a significantly tighter K_D_ of ~ 26 pM, affirming this interaction as the strongest currently known for a CaM:substrate binding. Following this, O’Donnell et al. used a variety of biophysical approaches to probe the binding of holo-CaM to the CaM binding region from βCaN and estimated a similar very tight K_D_ of ~1pM [48]. The affinity of holo-CaM for CaN appears to be the result of very rapid association (4.6 × 10^7^ M^− 1^ s^− 1^) and very slow dissociation (0.0012 s^− 1^) in the presence of saturating calcium concentrations.

Cook and Creamer employed stopped-flow fluorescence to explore the basis for the rapid binding of holo-CaM to αCaN [49]. In this work the binding of CaM to a peptide corresponding to the CaM binding region (pCaN), a construct consisting of just the regulatory domain, and full-length αCaN was studied. These authors found that at 37 °C the binding affinity is almost entirely due to direct interactions between the CaM binding region and holo-CaM, and that the association rate was sensitive to salt concentration, becoming faster at lower salt concentrations. This latter observation suggested that the association was in part driven by electrostatic interactions, not surprising given holo-CaM is very acidic and the CaM binding region in αCaN basic. What was noteworthy was the distribution of charged groups within the disordered regulatory domain, but outside the CaM binding region, appeared to play a role in determining the rate of association [49]. Computer simulations of the regulatory domain suggested that the distribution of charges resulted in the N-terminal half of the regulatory domain, which contains the CaM binding region, is more extended than the C-terminal half. This would result in the CaM binding region being accessible to holo-CaM while potentially minimizing unproductive interactions with the C-terminal half of the regulatory domain.

Overall it appears that CaN has evolved to bind rapidly and very tightly to holo-CaM. Indeed this appears to be the tightest CaM:substrate interaction reported to date.

#### A conundrum

The studies described above of the binding of holo-CaM to αCaN have revealed an interesting conundrum: why has the regulatory domain of CaN evolved to bind very rapidly and tightly to holo-CaM and also to have a functionally-important distal helix that is not very stable, potentially dynamic, at physiologic temperature? It is of course impossible to know the answer to this, but it is possible to make some educated speculations.

The very tight binding of holo-CaM to αCaN may be an indication of the physiologic importance of the phosphatase. It has been suggested that CaM levels in human cells are limiting [42]. In other words, there are fewer CaM molecules than there are substrate molecules. The tight binding of holo-CaM to αCaN would ensure the phosphatase takes precedent, becoming activated even in the presence of other CaM substrates. The rapid association could also allow for rapid coupling of increased calcium levels to phosphorylation states, likely ensuring that binding of CaM to αCaN is not a rate-limiting step in associated signaling processes. If this speculation is correct, then the overall activity of CaN should not be significantly affected by pathogenic mutations recently identified in CaM since these occur in just one of three identical genes that code for the protein [102–106]. Whether that holds true has yet to be seen.

A second potential reason for the very rapid association between CaM and CaN was discussed in an earlier review on activation of the phosphatase [34]. In that review it was suggested that the regulatory domain was in fact only transiently disordered. In short, the regulatory domain is thought to be folded in the inactive state of CaN (Fig. 3). When intracellular calcium levels rise CnB binds the metal ion allowing its N-terminal lobe to bind to the B-chain binding helix of αCnA and releasing the regulatory domain into a disordered state. Meanwhile, CaM has also bound calcium and can rapidly bind to the newly disordered regulatory domain resulting in the later domain folding into a different conformation and activating the phosphatase. The regulatory domain is thus disordered for just a short period of time. The disorder is important in that it could facilitate the rapid binding of CaM by making the binding site more accessible and leading to rapid activation [34]. Rapid activation of CaN would be important in some of its signaling roles. For example, channels are often open and close quickly (e.g. AMPARs [107]), requiring enzymes such as CaN that modulate their activity to function rapidly.

As suggested previously, the weakly stable, potentially dynamic, distal helix may have evolved to allow for fine control of CaN activity in response to increased body temperature (e.g. due to fever) [90]. This would allow signaling processes such as those associated with inflammation to be attenuated in response to fever. Unfortunately, this hypothesis would be difficult to test. For example, raising the temperature of cultured human cells will result in numerous intracellular responses complicating efforts to isolate the affects upon CaN alone.

## Conclusions

This enzyme is clearly a vital component of multiple signaling pathways and yet there is still much to learn about CaN. Our knowledge of the activation process has gaps including the structures of the inactive state and the holo-CaM:CaN complex. The evolutionary reasons for holo-CaM’s affinity for CaN being so high and yet the functionally vital distal helix formed upon binding being just marginally stable are matters for speculation. Furthermore, it is not clear that we understand how some substrates, e.g. tau, bind to CaN. Despite having been first discovered over 40 years ago, there is still much to learn about CaN [108–110].

## Data Availability

Not applicable.

## Abbreviations

CaM: Calmodulin
CaN: Calcineurin
CnA: Calcineurin A chain
CnB: Calcineurin B chain
NFAT: Nuclear factors of activated T-cells
FOXO: Forkhead transcription factors
MEF2: Myocyte-specific enhancer factor 2
TFEB: Transcription factor EB
AMPAR: α-amino-3-hydroxy-5-methyl-4-isoxazole-propionic acid receptor
NMDAR: NMDA-type ionotropic glutamate receptor
TWIK: Tandem-pore-domain weakly inward rectifying potassium channel
TRESK: TWIK-related spinal cord potassium channel
NHE1: Na+/H+ exchanger 1
DRP1: Dynamin-related protein 1
BAD: Bcl-2/Bcl-XL-antagonist, causing cell death
PDB: Protein databank
HXMS: Hydrogen-deuterium exchange mass spectrometry
NMR: Nuclear magnetic resonance
FTIR: Fourier-transform infrared spectroscopy
CD: Circular dichroism spectrapolarimetry
Rcan1: Regulator of calcineurin 1
